# Significance of EEG-electrode combinations while calculating filters with common spatial patterns

**DOI:** 10.3205/000334

**Published:** 2024-09-25

**Authors:** Dominik Wetzel, Paul-Philipp Jacobs, Dirk Winkler, Ronny Grunert

**Affiliations:** 1 University of Applied Sciences Zwickau, Faculty of Physical Engineering/Computer Sciences, Zwickau, Germany; 2 University Leipzig, Department of Diagnostic and Interventional Radiology, Leipzig, Germany; 3 University Leipzig, Department of Neurosurgery, Leipzig, Germany; 4 Fraunhofer Institute for Machine Tools and Forming Technology, Fraunhofer Plastics Technology Center Oberlausitz, Zittau, Germany

**Keywords:** algorithms, electroencephalography, statistics

## Abstract

**Objective:**

Common spatial pattern (CSP) is a common filter technique used for pre-processing of electroencephalography (EEG) signals for imaginary movement classification tasks. It is crucial to reduce the amount of features especially in cases where few data is available. Therefore, different approaches to reduce the amount of electrodes used for CSP calculation are tried in this research.

**Methods:**

Freely available EEG datasets are used for the evaluation. To evaluate the approaches a simple classification pipeline consisting mainly of the CSP calculation and linear discriminant analysis for classification is used. A baseline over all electrodes is calculated and compared against the results of the approaches.

**Results:**

The most promising approach is to use the ability of CSP to provide information about the origin of the created filter. An algorithm that extracts the important electrodes from the CSP utilizing these information is proposed.

The results show that using subject specific electrode positions has a positive impact on accuracy for the classification task. Further, it is shown that good performing electrode combinations in one session are not necessarily good performing electrodes in another session of the same subject. In addition to the combinations calculated using the developed algorithm, 26 additional electrode combinations are proposed. These can be taken into account when selecting well-performing electrode combinations. In this research we could achieve an accuracy improvement of over 10%.

**Conclusions:**

Carefully selecting the correct electrode combination can improve accuracy for classifying an imaginary movement task.

## Zusammenfassung

**Ziel:**

Common Spatial Patterns (CSP) ist eine gängige Filtertechnik, die für die Vorverarbeitung von Elektroenzephalographie-Signalen (EEG) zur Klassifizierung gedachter Bewegungen verwendet wird. Besonders in Fällen, in denen nur wenige Daten verfügbar sind, ist es wichtig, die Anzahl der Merkmale zu reduzieren. In dieser Forschungsarbeit werden verschiedene Ansätze zur Reduzierung der Anzahl der für die CSP-Berechnung verwendeten Elektroden untersucht.

**Methoden:**

Frei verfügbare EEG-Datensätze werden für die Evaluierung genutzt. Dazu wird eine einfache Klassifizierungspipeline bestehend aus CSP und linearer Diskriminanzanalyse zur Klassifizierung genutzt. Es wird eine Basislinie unter Nutzung aller Elektroden berechnet und diese mit den Ergebnissen der verschiedenen Ansätze verglichen.

**Ergebnisse:**

Der vielversprechendste Ansatz besteht darin, die Fähigkeit von CSP zu nutzen, Informationen über den Ursprung des erstellten Filters zu liefern. Es wird ein Algorithmus vorgeschlagen, der diese Fähigkeit ausnutzt und so wichtige Elektroden aus dem CSP extrahiert.

Die Ergebnisse zeigen, dass die Verwendung von probandenspezifischen Elektrodenpositionen eine positive Auswirkung auf die Genauigkeit der Klassifizierungsaufgabe hat. Außerdem wird gezeigt, dass Elektrodenkombinationen, die in einem Versuch gut funktionieren, nicht unbedingt auch in einem anderen Versuch desselben Probanden gut abschneiden. Zusätzlich zu den mit Hilfe des entwickelten Algorithmus berechneten Kombinationen werden 26 weitere Elektrodenkombinationen vorgeschlagen. Diese können bei der Auswahl gut funktionierender Elektrodenkombinationen berücksichtigt werden. In dieser Arbeit konnten wir damit eine Verbesserung der Genauigkeit von über 10% erzielen.

**Schlussfolgerung:**

Die sorgfältige Auswahl der richtigen Elektrodenkombination kann die Genauigkeit bei der Klassifizierung von einer gedachten Bewegung verbessern.

## Introduction

Common spatial pattern (CSP) is an algorithm which decomposes a signal into spatial patterns that are extracted from multiple classes. These are used to calculate spatial filters that maximize the ratio of the variance of one class to another [1], [2]. It can be utilized to decompose an electroencephalography (EEG) signal into components that separate different classes [1], [2], which in turn can be used to control a brain-computer interface (BCI). BCIs can be used to control different types of hardware and software, such as wheel chairs [3] or an avatar in a virtual reality environment [4]. Integration of BCIs into the rehabilitation therapy of stroke patients is an ongoing research subject [5], [6].

Many variants of CSP exist and are still subject to research [7], [8], [9]. As CSP is used to create signal-specific filters; an interesting characteristic of CSP is the ability to reveal information about the origin of the created filter. This can be utilized to select important features or electrodes for a specific subject [10], [11]. Wang et al. calculate the event-related desynchronization and readiness potential using all electrodes and select only those electrodes that have maximum value based on the computed CSPs [10]. Another approach is omitting channels where the average of the CSP mixing matrix coefficients is less than a certain threshold and evaluate the remaining signals with the help of a neural network [11]. However, the authors did not state the impact on the electrode reduction and did not compare it with other approaches.

In this study, our objective is to identify improved electrode combinations for classifying an imaginary movement (IM) task through the use of CSP. We aim to compare these combinations with CSP calculations incorporating all available electrodes.

## Material and methods

In this work two different approaches are proposed:

- Approach 1: Check every possible combination on one dataset, take the best combinations and try them on the other datasets (to see if some generalization is possible).
- Approach 2: Infer possible combinations for each subject in each dataset using the patterns from the base CSP calculation.

Another approach would be to try every possible combination for each subject in each dataset and choose the best one. Due to the high computational expense this approach is unfeasible and therefore was discarded.

**Figure 1:**
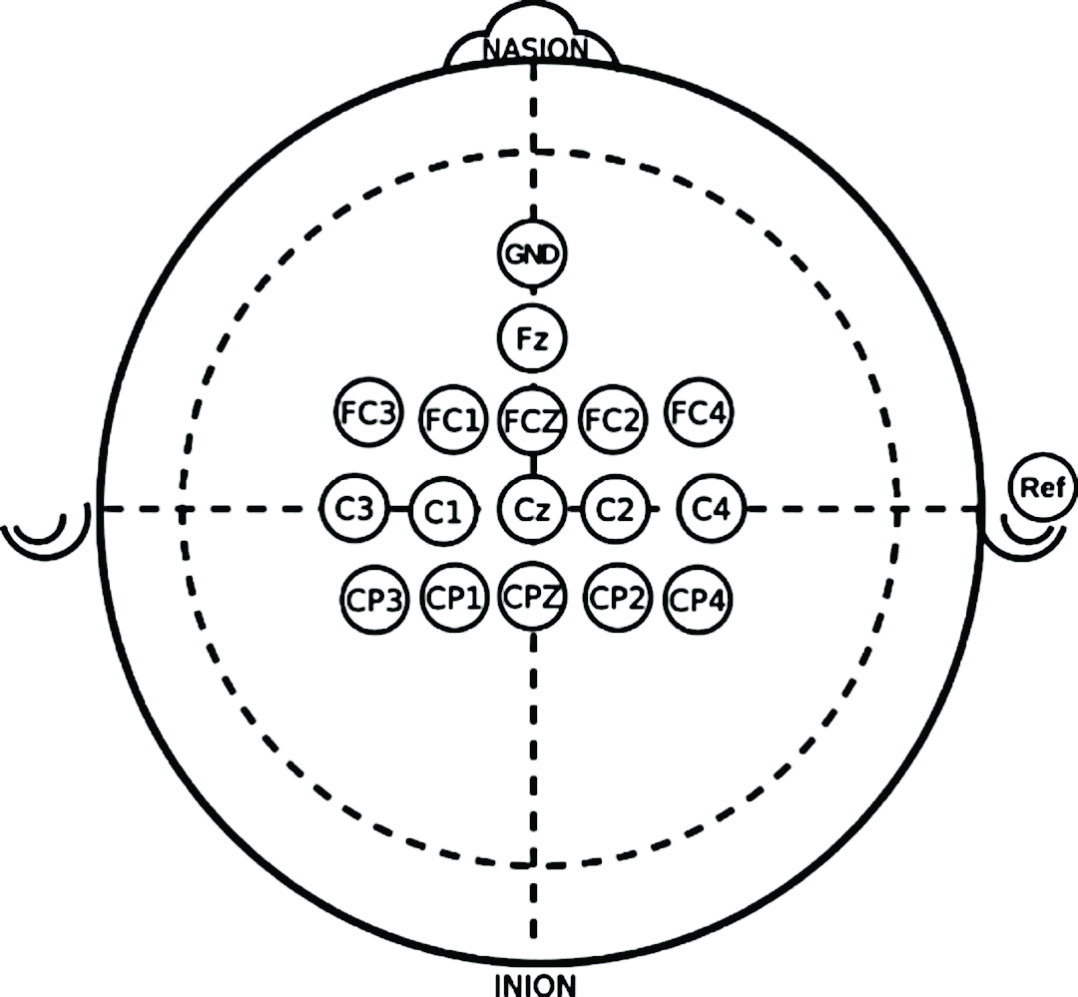
The electrode positions used for pre-evaluation Figure by Wetzel et al. [13], licensed under CC BY 4.0 (https://creativecommons.org/licenses/by/4.0/), and adapted from Oxley [26], licensed under CC0 1.0 Universal (https://creativecommons.org/publicdomain/zero/1.0/)

To acquire the needed data we utilize the library “Mother of all BCI Benchmarks” (MOABB) [12] as it contains interfaces to different freely available BCI datasets. Only datasets that fulfill the following conditions were considered for the evaluation:

- All sessions in the dataset are recorded with at least 90 Hz sampling rate.
- The paradigm of the dataset contains at least a left-hand imagination and a right-hand imagination class.
- The electrodes used for data acquisition contain at least the electrodes used in [13] (see Figure 1 (Fig. 1)).

Four datasets meet these conditions: Yi2014 with n=10 [14], BCI Competition IV Dataset 2a (BNCI2014) with n=9 and 2 sessions per subject [15], Cho2017 with n=49 [16] and PhysioNet with n=109 [17].

**Figure 2:**
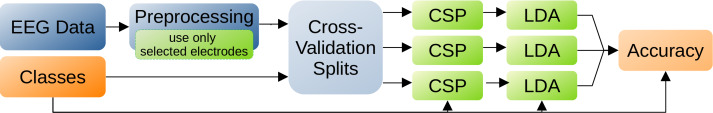
Single pipeline for calculating the accuracy

We use the EEG data from the datasets with the left-hand imagination and right-hand imagination classes (which consist of single finger or full hand movements). Subsequently we utilized a simple pipeline as shown in Figure 2 (Fig. 2) containing: 1) a preprocessing step, where the data is reduced to the specified electrodes and bandpass filtered between 5 and 45 Hz, 2) predefined cross-validation splits, 3) calculation of the CSP filter for each split and filtering the signal with it using a CSP implementation from MNE-Python [18], 4) a classification step with linear discriminant analysis (LDA) and 5) the combination of the accuracies to a mean accuracy. 

**Baseline:** We perform a baseline calculation with the pipeline utilizing all electrodes for each subject from the dataset (BFull) as well as the pipeline with 16 electrodes that are positioned as shown in Figure 1 (Fig. 1) (B16).

**Approach 1:** We consider only the 16 electrodes leading to an amount of possible combinations of 
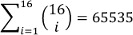
. We choose to only use combinations that contain at least 8 electrodes to have enough features for LDA after the CSP filter, which results in 
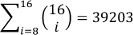
 combinations. Further we decided to use Yi2014 as base dataset as it is fast to calculate for each combination. We run the pipeline for all combinations on each subject of Yi2014. The percentage change between the 16 electrode baseline accuracy (of Yi2014) and each combination’s accuracy is calculated afterwards. Last, we assess whether the expectation µ is significant better over all subjects for each combination using a t-test with the following hypothesis:

(1) H_0_:µ≤0, H_a_:µ>0 and α=5%

Significant combinations are used for processing the other datasets. 

**Figure 3:**
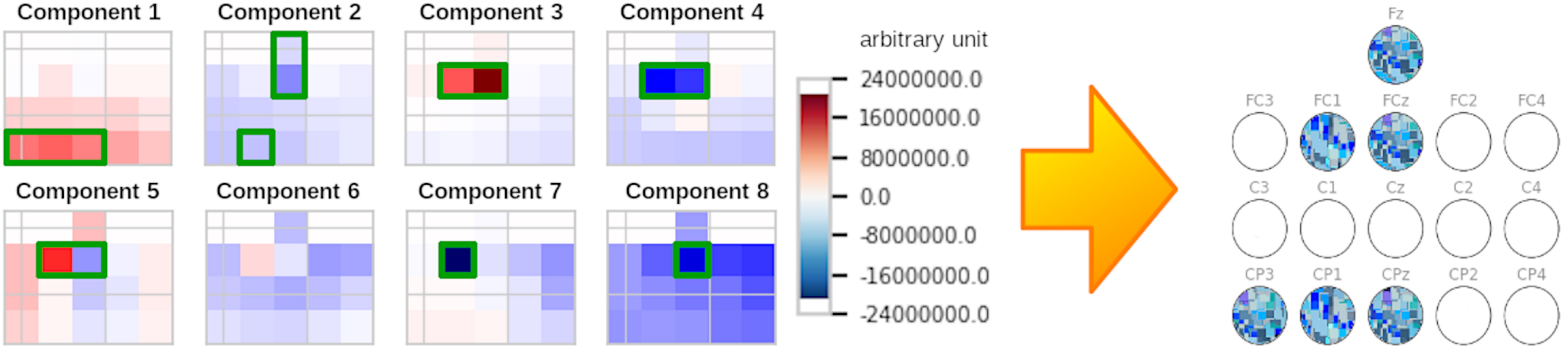
The 8 components of the baseline of one subject of Yi2014, with the selected electrodes for each component with green border. The extracted electrode combination is Fz, FC1, FCz, CP3, CP1, CPz.

**Approach 2:** We developed an algorithm to infer more useful electrodes by using the CSP calculation from the baselines. Therefore, we use the pattern p from the first 8 CSP components [the amount of components calculated with CSP is the amount of electrodes, but we only consider the first 8 as the latter are less significant] and select the electrodes that fulfill the following condition:

(2) |e_p_–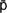
|>θ·σ(p)

where e_p_ is the value of the electrode in the pattern p, 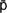 is the mean of all electrode values of the pattern, θ is a predefined threshold and σ(p) is the standard deviation of all electrode values of the pattern. In our experiments we set θ=1.5. Figure 3 (Fig. 3) shows a visualization of the algorithm.

**Figure 4:**
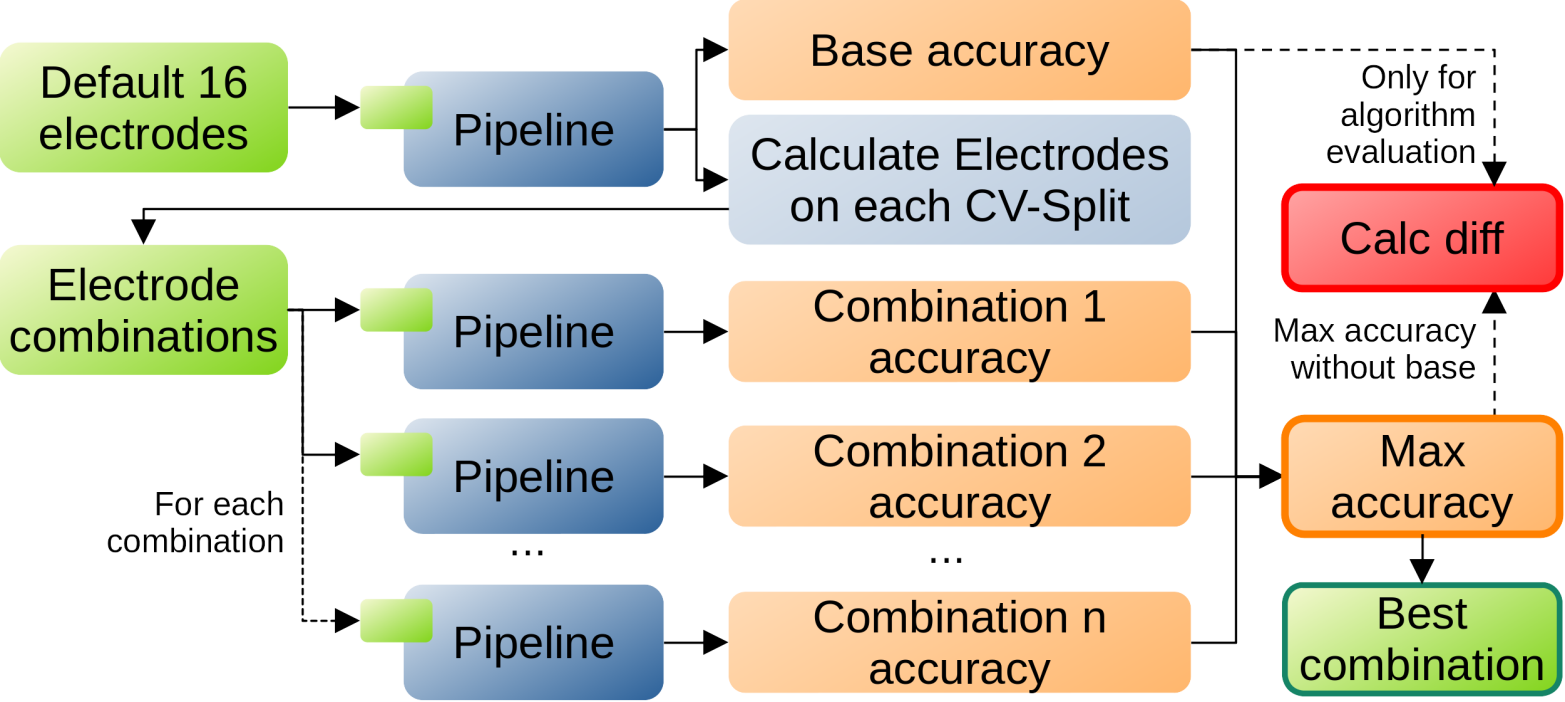
The complete pipeline for approach 2 to calculate the best electrode combination

We calculate the electrodes for each cross validation split. The found electrode combinations (containing more than 2 electrodes) are then used to calculate the accuracy for the subject and the combination with the highest accuracy is furthermore compared against the baselines. The complete pipeline is visualized in Figure 4 (Fig. 4). 

## Evaluation

To evaluate our approaches we perform Bayesian A/B tests [19], [20]. We compare different variants against the full electrode baseline (BFull). The used variants are:

- The 16 electrode baseline (B16)
- The best electrode combinations from approach 1 (A1)
- The per subject best electrode combinations using our algorithm with 16 electrode base (Algo16)
- The per subject best electrode combinations using our algorithm with full electrode base (AlgoFull)
- The per subject best electrode combinations of the combinations from approach 1 (PSA1)
- The per subject best electrode combinations of the combinations from approach 1 and our algorithm (both bases) (Comb)

We use the beta distribution for our prior and posterior distribution as we have dichotomic data. 

Further we assume a priori that all variants perform better than average so we set α=6 and β=4. However the prior has a marginal effect due to the amount of samples provided. Afterwards we perform a Monte-Carlo simulation with n=10,000 for our variants and calculate the fraction of each variant against the full electrode baseline. By calculating the mean of these fractions we get a percentual improvement against BFull.

## Results

After testing all combinations on Yi2014, 26 remain as significant over all 10 subjects from Yi2014. Applying those combinations separately on the other datasets did not lead to improvements of the accuracy compared to BFull (see Table 1 (Tab. 1) and A1 in Table 2 (Tab. 2)), whereas using only the best of all 26 combinations for each subject individually increases the accuracy significantly (see PSA1 in Table 2 (Tab. 2)). Using the proposed algorithm improves accuracy compared to BFull, but it is not as effective as PSA1 (see Algo16 and AlgoFull in Table 2 (Tab. 2)). Interestingly combining the different approaches leads to an improvement of more than 10% (see Comb in Table 2 (Tab. 2)).

**Figure 5:**
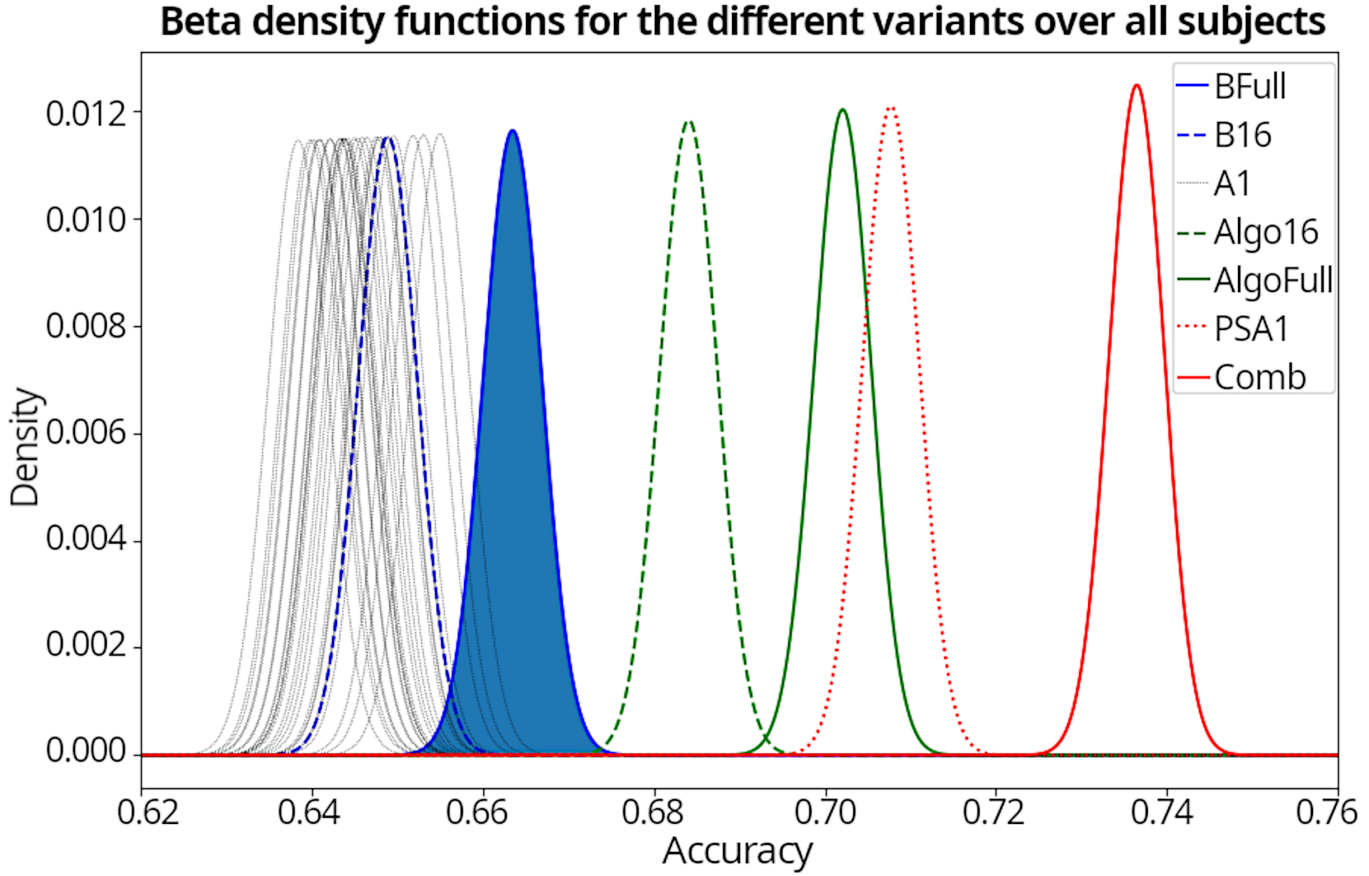
The beta density functions for all variants over all subjects. The black lines (A1) show the beta distributions from the 26 selected combinations. The curve filled in blue is the baseline using all electrodes, which is also the base for comparison.

These findings are also visualized in Figure 5 (Fig. 5), which shows the computed beta distributions of where the samples are taken from for the percentual improvement calculation.

**Figure 6:**
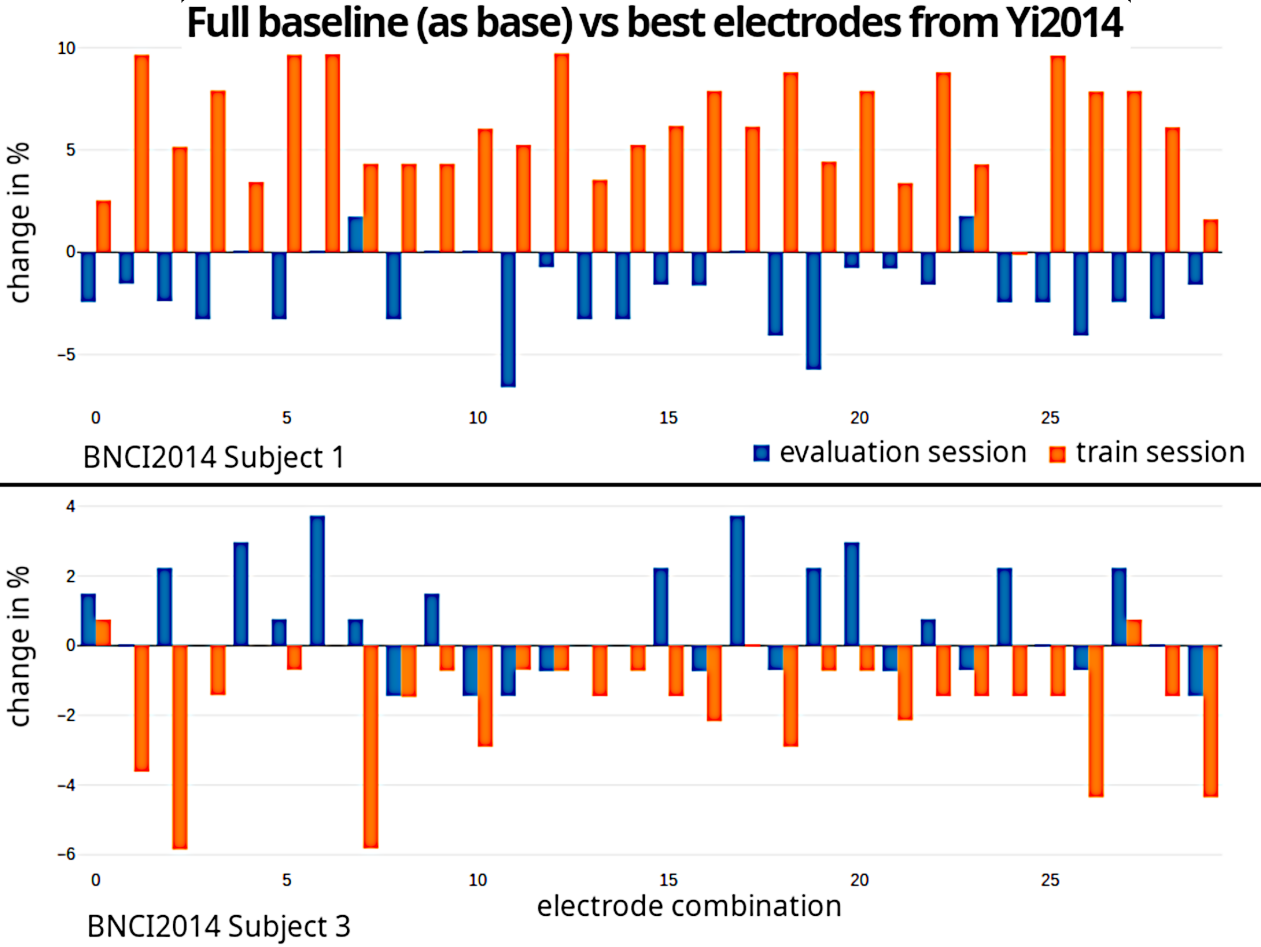
The percentage change using a combination compared to full baseline. Upper image is subject 1 and lower image is subject 3 of the BNCI2014 dataset. One pair of blue and orange bars correspond to one electrode combination.

As BNCI2014 contains 2 sessions per subject, we test whether a good performing combination in one session also improves the accuracy for the other session. Figure 6 (Fig. 6) shows the result for subject 1 and 3. It is obvious that a combination which works well for one session may not perform equally well in another session. 

## Discussion

Our results show that using only specific electrodes has the potential to increase the accuracy of an IM task compared to using all electrodes (as also shown in [21]). Reducing the amount to a predefined number of electrodes (from all to 16) does not lead to better results. We also showed that even for the same subject the calculated electrode combination cannot be transferred to another session. In summary, it can be seen that it is important to calculate a well-performing electrode combination for each session and subject so that the overall amount of electrodes applied to the subject cannot be reduced, but the evaluation speed and accuracy of an IM task can. The proposed algorithm for determining good performing electrode combinations can be used even in a clinical setting, as it is fast to calculate and EEG tasks usually need calibration anyway. Furthermore, we proposed 26 electrode combinations that can be considered additionally for better performance. In summary, sophisticated selection of the appropriate electrode combination can enhance the accuracy of an IM task. In our research we achieved an improvement of over 10%.

To further improve performance the algorithm to detect electrode combinations could be revised or changed as there are subjects where it performs worse. It was assumed that this is due to a low amount of channels. However, there is no correlation between the number of channels and the percentage of change. Another step could be to determine another set of well-performing electrode combinations using the brute-force calculation (approach 1) on another dataset and compare it to the shown results. 

## Notes

### Source code

The source code for this research is available at https://gitlab.com/domwet/csp-research.git. 

### Datasets

The underlying datasets can be accessed as follows:

- Yi2014 [14]: https://doi.org/10.7910/DVN/27306 [22]
- BNCI2014 [15]: https://doi.org/10.21227/katb-zv89 [23]
- Cho2017 [16]: https://doi.org/10.5524/100295 [24]
- PhysioNet [17]: https://doi.org/10.13026/C28G6P [25]

### Competing interests

The authors declare that they have no competing interests.

## Figures and Tables

**Table 1:**
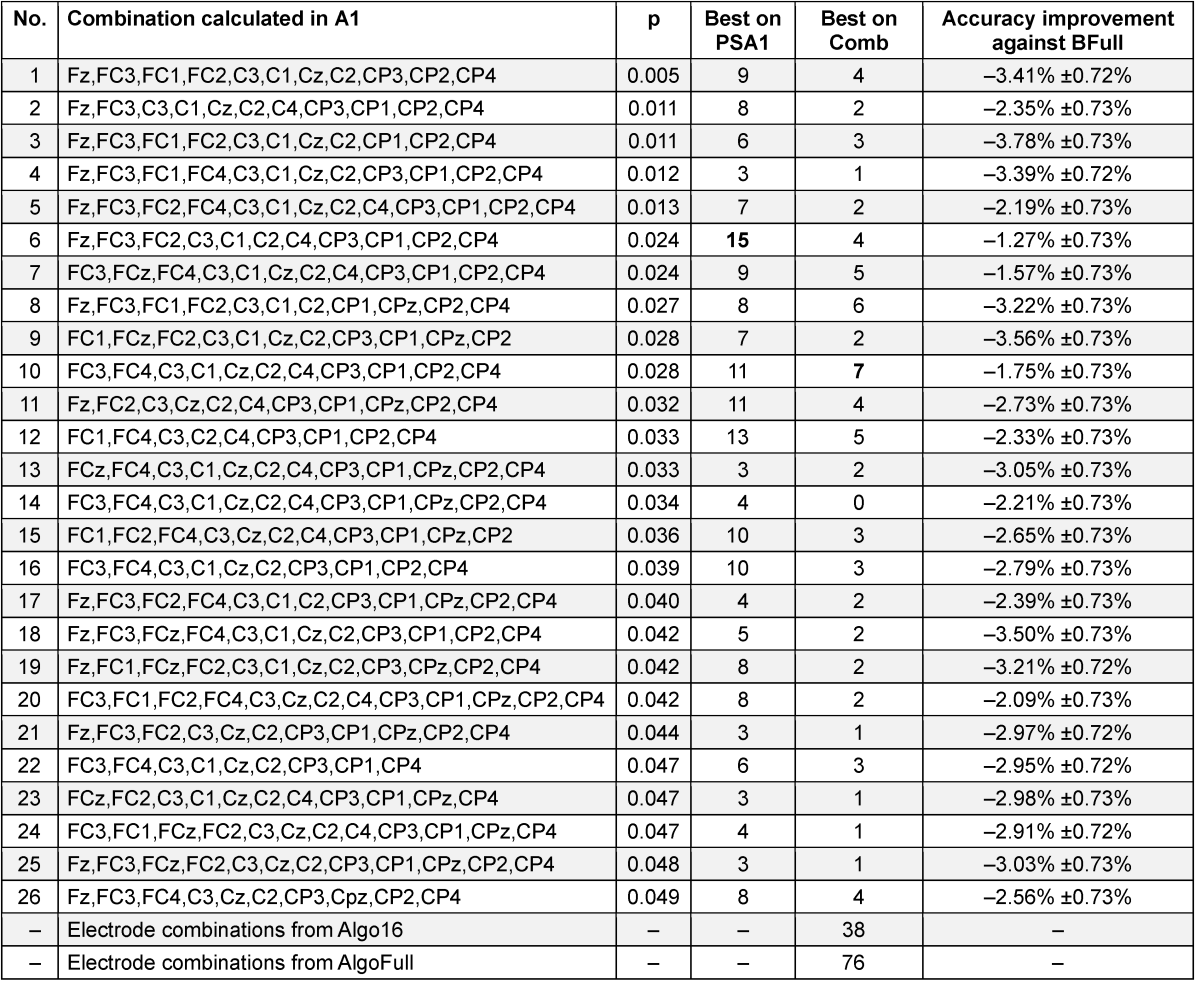
The combinations that where selected with approach 1 (and their p-values) and how often each combination is considered the best across the different variants (Best on PSA1 and Best on Comb) as well as the performance against the full baseline using each combination for all subjects (Accuracy improvement against BFull). Bottom lines show how frequently combinations from our algorithm were selected on Comb.

**Table 2:**
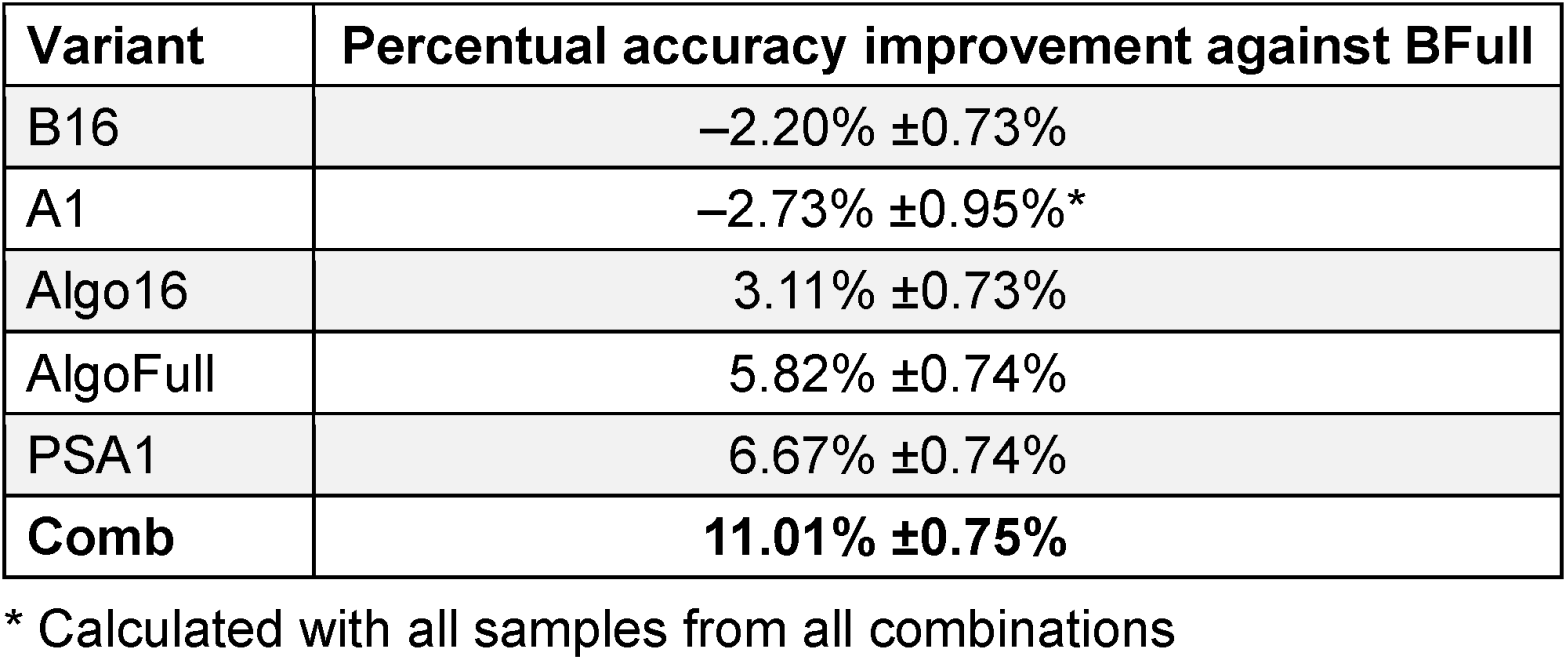
The performance of the different variants against BFull (Best bold)
